# Antisepsis in Gynecology

**Published:** 1888-05

**Authors:** S. Pozzi

**Affiliations:** Fellow-Surgeon to Lourcine Pascal Hospital


### SELECTIONS FROM THE FRENCH.

### ANTISEPSIS IN GYNECOLOGY.*

BY DR. S. POZZI, FELLOW-SURGEON TO LOURCINE PASCAL HOSPITAL.

Translated by F. J. Arbeely, A. B., M. D., Atlanta, Ga.

GENTLEMEN—All adopted rules of antisepsis in general surgery are applicable to gynecology, but there are some particular details and interesting methods to which I would like to draw your attention. To make my remarks clear, I will divide my subject into two paragraphs:

First—In relation to all operations made on the vagina, neck of the uterus and uterine cavity through the natural canal; and

Second—To those operations which are made through the abdominal walls.

1. Let us examine successively the antisepsis concerning the operator, instruments and patient.

A. *Operator.*—We all know the importance of absolute cleanliness of the hands in operative surgery; but there is more importance attached to those cases in which we have to deal with the interior of cavities, where the deposited germs may find their way to a good soil, essentially favorable for their culture, pullulation and rapid development of infection, as in the uterine and vaginal cavities.

The finger-nails must be cleaned carefully and the arms well washed up to the elbows. The pulverized almond, so extensively used in the midwifery wards to clean the hands, is nearly always infected with germs, coccus and bacilli of all sorts; therefore it must be absolutely banished. All kinds of soaps are good and useful, except the hard soap, commonly known as Savon de

*Read before the Faculty of Medicine of Paris.

Marseille, which, on account of the low temperature and the impurity of the tallow generally used in its preparation, gives clear evidence of infection. This is what Dr. Von Eiselsberg's observation has shown in his experiments on the different agents frequently used in cleansing the hands in hospital practice in Professor Billroth's Clinic.

The hands, having been cleansed with soap, we must wash them with $\frac{1}{1000}$ corrosive sublimate solution; some operators, however, prefer plunging the hands and arms first in $\frac{1}{1000}$ permanganate of potash solution, which colors the skin violet-brown, but this disappears when it is washed with saturated solution of oxalic acid. I believe this is unncessary, and should be practiced only in those exceptional instances when the operator has been mingling with septic matter, or where he has suspected the same previously.

When we are called to deal with fetid matter (as in cases of uterine cancer, etc.), it is very important that we should use some sort of deodorizer with the antiseptic, or the hands of the operator become impregnated with the disagreeable odor in spite of all other means used to guard against this inconvenience. Foulis (of Edinburg) recommends the immersion of the hands in the essence of turpentine—a vessel containing $\frac{1}{1000}$ corrosive sublimate solution should be placed near by, and in such manner as to allow the operator to dip and wash his soiled hands in it frequently.

B. *Instruments.*—The more simple the instrument the better it is for use. An instrument made out of one piece is much better than the one which is composed of many pieces forming several cavities, joints, grooves, etc., unless it is constructed in such manner as to be easily detached, cleaned and well disinfected; therefore the slip-knots, hysterometers, the needle-holder forceps, which is provided with spring, the canulated needle generally used for sutures, even the movable eye needle of Reverdin, who has given its first model, with its great convenience, must be abandoned.

As it is very essential to leave the instruments, immediately after an operation, in boiling water for some time, it is also equally important to keep them in $\frac{50}{1000}$ carbolic acid solution for at least half an hour before the next. The reason why I do not advise the corrosive sublimate is because of its destructive power upon the metals. To guarantee the absolute cleanliness of an instrument previously used in a septic subject, fetid pus, purulent or gangrenous matter, etc., it must be first exposed to 110 degrees heat in an oven, or kept in the boiling carbolic acid solution above mentioned for fully half an hour or an hour, or two, if it is cold. Although some material alteration might take place in the construction of the instruments, especially in the bistouries, in going through such process, this is absolutely necessary and it must be done.

C. *Patient.*—In antisepsis of the external genito-organs and of the vagina, the patient should take a bath (the sublimate in preference) the evening before the operation, or the same morning, the bowels emptied carefully by an enema. The operator, or his assistant, before cleansing his hands, draws the urine and shaves the labium and adjoining parts to prevent the possibility of septic matter which often lodges there and renders the operation unsuccessful.

To clean the external genito-organs well, we should use the soap and brush first, and the bichloride of mercury solution (1 to 1000) next. In washing the vagina we can use the same solution diluted with half its amount of hot water.

Certain physicians have lately doubted the safety of the sublimate solution in gynecology, especially in obstetrics. It is true, that when this agent was first introduced to the profession, it was carelessly used without discrimination, and for this reason, perhaps, the reaction was slow. I consider myself that a $\frac{1}{2000}$ solution as a vaginal injection, when properly used, is very safe and without the least danger whatever. Many works have been published on this subject, but rarely any give the details or touch the point which indicates the differences existing between the use of these injections shortly after delivery and those which are used in some other conditions. In newly-delivered women we all know that the vaginal and uterine cavities are widely connected by an intermedial cervix, more or less soft and open; consequently, if an injection thrown carelessly into the vagina passes easily to the uterine cavity, where it accumulates, remains and might be absorbed by coming in contact with the flabby and freshly detached mucous membrane surface, especially if the vaginal walls are not kept separated by the fingers during the application and the return of the fluid. This is the reason why so many accidents occurred from the use of such simple vaginal injection, which has led some operators to doubt the safety of the drug. I do not base my observations only on those experiments which have been made on the doe rabbits or on the females of the Indian hog, as they are hardly enough to demonstrate this special point.

Let me remind you here of the fact that as soon as the sublimate solution ordinarily used comes in contact with a profuse secretion, whether leucorrheal or cancerous ichor, etc., it is rapidly neutralized and loses its toxic and disinfectant power to a great extent. Ernest LaPlace has shown recently the unreliability of this antiseptic, discovered the cause and how to remedy it. It seems that this mercurial salt loses its antiseptic effect in the presence of an albuminoid by forming albuminate which precipitates rapidly. His experiments were as follows: To an open glass tube containing $\frac{1}{25}$ cubic cent. of natural serum he added 5 cubic cent. of $\frac{1}{1000}$ sublimate solution without any effect on the germ development; when this quantity was added to ½ cubic cent. of serum the bacteria remained unchanged; then when 5 cubic cent. of $\frac{1}{1000}$ sublimate solution were added to ⅛ of a cubic cent. of putrefied human blood containing bacteria, the microbes were still increasing; and on cultivating a few drops of the mixture on gelatine, according to Esmarch's method, in five days colonies of staphylo-coccus were plainly noticed. LaPlace observes also that the addition of $\frac{5}{1000}$ of tartaric acid to the sublimate solution would render it acid, and by this process he arrests the formation of mercurial albuminate. In repeating the above-mentioned experiments to assure this procedure, he could not discover the least sign of germ development. I consider this a very important discovery to general surgery, especially to gynecologists. I am practicing it and cannot speak too highly of its merits.

Let me point out here certain practical rules which might appear to you very common and simple, but I consider them very important in this connection. The irrigator is a bad instrument and it must be abandoned. To clean the vagina properly we must follow certain rules. Washing the vagina, which I generally call rinsing, is very necessary and every surgeon must practice it before operating. The following apparatus is my preference and will answer all purposes: A receiver, provided with a tube near the bottom, armed with a glass canula, which could be easily detached and disinfected, near the end. When an injection is required put the receiver on a moderately elevated place. Introduce the canula carefully between the medius and the index, push it in slowly to the cul-de-sac and let the solution run in; every now and then turn the canula around in the vagina in such a manner as to allow the wash to come in contact with all parts and between all folds in the cavity, or some unclean place may remain untouched and cause infection. The patient must be ordered to use these injections, followed by a tampon of iodoformed gauze after each one, applied to the vagina, for a week or more before the proposed operation.

In case where the affection is associated with an offensive odor, as, for instance, in cancerous vegetation, gangrenous fibrous tumor, etc., some sort of deodorant wash (which has an antiseptic power at the same time) must be used first. Labarraqùe's quis solution, or Pennis vinegar diluted with hot water, will answer the purpose admirably.

A few words concerning iodoformed gauze will not be out of place. The commercial gauze commonly used contains from 20 to 30 per cent. of iodoform; for hospital use, it is more econominal and much safer when it is prepared personally or by persons whom we know well. Impregnate a piece of the gauze ten yards long, or eight pieces one yard each, in the following solution:

| | |
|---|---|
| Iodoform | 50 grams. |
| Glycerine | 100 grams. |
| Alcohol | 700 grams. |

Suspend in the air until they are dried; put in an air-tight can until needed for use.

There are some curious facts I desire to mention here, in which Dr. Von Eiselberg's experience in Billroth's clinic shows that the gauze still contained some germs (11 in 30) and could be easily cultivated, notwithstanding it was prepared according to the best method and with the utmost care ; but when it was exposed first to a temperature of 100 degrees (easily done by boiling), this culture was unsuccessful in 18 out of 20 cases; therefore to destroy all germs and seeds and to leave no doubt whatever, it is best to heat the gauze in an oven or such apparatus even to 120 degrees. But this is practically very inconvenient in some instances, and the sterilization in boiling water will be sufficient and will answer the purpose.

You may be astonished to hear the statement that the use of iodoform is not sufficient to neutralize the germs, but you must remember the experiments of Heyn and Rosving by which they had shown plainly that the iodoform in vitro is not a germicide, not even an obstacle in the way of germ development. Dr. DeRuyter (of Berlin) came to the same conclusion and accepted C. B. Tilanus' views in his recent experiments—that is, the iodoform in vivo is not antiseptic where there is pathogenic ferment. The solution of this seemingly contradictory law, according to Dr. Behring's observations, is this: the iodoform reduces the leucomaines and ptomaines as soon as they are formed, and thus destroys them whenever they are present.

ANTISEPSIS OF THE NECK AND UTERINE CAVITY.—After all operations made on the uterus or the neck, it is best to have some sort of an antiseptic to remain in the cervical canal. I am in the habit of leaving a suppositorium made according to the following formula:

℞.—Iodoform .......................... 20 grams.

Glycerin......................... }

Gum Arabic..................... } *àà* 2 grams.

Starch ........................... }

These suppositories have the advantage of being easily handled and pushed forward into the uterus, but sometimes they are incompletely dissolved, causing colic. This only disadvantage led me to abandon their use and replace them by sprinkling some pulverized iodoform on the neck or leaving a tampon of iodoformed gauze in the cervix.

The most important part of our subject is to know how to disinfect these agents commonly used for the dilatation of the os uteri. I consider the tupelo and prepared sponges as inferior agents, and much prefer the laminaria; the reason that the latter is the cause of infection in some cases is simply from the want of more precaution on the part of the surgeon.

There are two ways to render those agents antiseptic, of which we can select one, the immersion in the concentrated solution of carbolic acid with alcohol (95 parts of carbolized alcohol to 5 of pure alcohol) which Martin adopted, or to let them remain while in the iodoformed ether, to which is added one-tenth of alcohol to prevent forming the caustic acid (Herff Darmstadt, Doléris, etc.); any one of the two we may choose. It is very important to wash the laminaria quickly with carbolic acid solution (20 to 1,000) or with the sublimate (1 to 1,000) before it is used.

There is much more danger from the use of these injections in the uterus in obstetrics than from those which are used in gynecology, except where the uterine cavity is very much dilated, or after an operation, where a large surface has been exposed and a big tumor cut out, for the condition of the uterus for absorption is the same as it is after delivery.

When the uterine cavity is not much dilated, the use of the sublimate (1 to 2,000) by means of a hard rubber double current sound is associated with no danger, but as most of the instruments employed are generally metal, I prefer the warm carbolic acid solution, (10 to 1,000), a quart or more being thrown into the cavity, until the liquid returns clear, which indicates that the intra-uterine cavity is sufficiently clean.

If an active antiseptic is needed in certain cases, as in gangrenous fibroma, intra-uterine cancer, with putrefied fungosity, etc., the injections of the sublimate (1 to 2,000) are preferable, but in cases where these injections have been frequently given, their use must be followed with some other irrigations which will assure the perfect evacuation of the toxic antiseptic. To accomplish this object, I recommend the irrigation with a solution of sea-salt (6 to 1,000), which will modify its endosmotic and irritant action by facilitating its composition with the serum of the blood. I use the sea-salt very frequently in cases where, from some reason or another, I have a difficulty in the use of antiseptics simply as an aseptic.

[TO BE CONTINUED.]

